# Music Therapy vs. Music Listening for Negative Symptoms in Schizophrenia: Randomized, Controlled, Assessor- and Patient-Blinded Trial

**DOI:** 10.3389/fpsyt.2021.738810

**Published:** 2021-12-21

**Authors:** Inge Nygaard Pedersen, Lars Ole Bonde, Niels Jørgensen Hannibal, Jimmi Nielsen, Jørgen Aagaard, Christian Gold, Lars Rye Bertelsen, Silvia Beatriz Jensen, René Ernst Nielsen

**Affiliations:** ^1^Department of Communication and Psychology, Aalborg University, Aalborg, Denmark; ^2^Aalborg University Hospital, Psychiatry, Aalborg, Denmark; ^3^The Music Therapy Research Clinic, Aalborg University, Aalborg, Denmark; ^4^Department of Psychiatry, Mental Health Centre, Copenhagen, Denmark; ^5^Department of Clinical Medicine, University of Copenhagen, Copenhagen, Denmark; ^6^Norwegian Research Centre (NORCE), Bergen, Norway; ^7^Department of Clinical and Health Psychology, University of Vienna, Vienna, Austria; ^8^Department of Clinical Medicine, Aalborg University, Aalborg, Denmark

**Keywords:** schizophrenia, negative symptoms, isolation, music therapy, music listening, assessor- and patient-blinded study

## Abstract

**Objective:** To investigate the efficacy of music therapy for negative symptoms in patients with schizophrenia.

**Methods:** Randomized, participant- and assessor-blinded, multicenter, controlled trial including patients diagnosed with schizophrenia according to ICD-10 with predominantly negative symptoms, between 18 and 65 years. Participants were randomized to 25 successive weekly individual sessions (excluding holidays, including cancellation by the participant) of either music therapy conducted by trained music therapists, or music listening together with a social care worker. The primary outcome was reduction in negative symptoms as measured by The Positive and negative Syndrome Scale (PANSS) negative subscale total score, assessed by a blinded rater, utilizing mixed-effects model analysis.

**Results:** In total, 57 participants were randomized; 39 completed the study's initial 15 weeks, and 30 completed follow-up at 25 weeks. On the primary outcome of PANSS negative subscale, no significant difference was observed between groups with a coefficient of −0.24 (95% CI −1.76 to 1.27, *P* = 0.754) in the intention to treat analysis, and −0.98 (95% CI −5.06 to 3.09, *P* = 0.625) when only analyzing completers. Both interventions showed significant reduction from baseline to 25 weeks on PANSS negative subscale. On secondary outcomes, no between group differences were observed in The Brief Negative Symptom Scale, WHOQOL-Bref (Quality of Life), The Helping Alliance Questionnaire and The Global Assessment of Functioning in the intention to treat or completers populations utilizing Mixed Effects Models.

**Conclusion:** No difference between groups randomized to music therapy vs. musical listening was observed resulting in no clear recommendation for which intervention to use as the first choice for treatment of patients diagnosed with schizophrenia and predominantly having negative symptoms.

**Clinical Trial Registration:** www.ClinicalTrials.gov, identifier: NCT02942459.

## Introduction

Negative symptoms in patients with schizophrenia have been correlated with lower quality of life, lower overall social and global functioning, as well as cognitive deficits (1). Several systematic reviews have shown that adjunct music therapy treatment is beneficial, useful and viable in general (2–4) and others have shown positive effect on negative symptoms (5–9). Music therapy is “a reflexive process wherein the therapist helps the client to optimize the client's health, using music experiences and the relationships formed through them as impetus for change” (10). This is different from music medicine and therapeutic music listening, where the carefully selected music itself is the agent of change (11). Both music therapy and music medicine vary in number of sessions and frequency and interventions are most often tailored to the need of the patient following a prior assessment. Music therapists in Denmark have a 5 year master's degree in music therapy. Research shows that <16 h of music therapy have a minor effect than 16 plus h (12). We estimated 25 h to be a maximum number of hours for the vulnerable population under investigation, while also meeting an effective minimum number based on previous findings (12). For both interventions in this study a manual was developed.

Another meta-analysis reported significant treatment effect of adjunct music therapy compared to treatment as usual (TAU) on positive and negative symptoms in patients suffering from schizophrenia (4). The review included 12 randomized controlled trials (RCTs) (*n* = 804), with authors concluding that patients who received music therapy improved to a larger degree than those who did not; in particular, patients with a more chronic course of illness experience effect (5). Another systematic review and meta-analysis investigating psychological and psychosocial interventions for negative symptoms in psychosis found that arts-based treatments were not effective in treating negative symptoms; however, a sensitivity analysis suggested benefit of music-based therapies compared with TAU (4). A third review investigating treatment interventions for negative symptoms in schizophrenia including both pharmacological and non-pharmacological treatments concluded no consistent evidence for an efficacy of any particular intervention for patients with negative symptoms. However, the authors suggested music therapy as a useful and viable intervention, along with pharmacological interventions, early treatment of psychosis and exercise, to reduce the severity of unspecified negative symptoms (4). Lastly, Jia et al. (9) included 18 studies comprising 1,212 participants comparing music therapy with control conditions utilizing meta-analytic methods. The analysis demonstrated that adjunct music therapy significantly improved total symptoms (SMD = −0.48, 95% CI: −0.74 to −0.22), negative symptoms (SMD = −0.56, 95% CI: −0.72 to −0.40), depressive symptoms (SMD = −0.35, 95% CI: −0.54 to −0.17), and quality of life (SMD = 0.35, 95% CI: 0.07–0.62) in people with schizophrenia, compared to controls. Controls received antipsychotic medication or psychiatric routine treatment solely. The sensitivity analysis supported the main results, however, authors concluded that quality of evidence was still low, i.e., more well-designed studies with larger sample size and high methodological quality are needed to confirm the efficacy of adjunct music therapy in treating patients with schizophrenia (9). In summary, the international evidence on music therapy for negative symptom reduction is mostly, but not conclusively, suggestive of beneficial effects (13); however, no study has been conducted in Denmark, and few studies have used active comparisons. None of the meta-analyses suggest explanations as to why music therapy might be beneficial. Some Danish and Norwegian case studies emphasize the freedom of improvisation in performing music—no specific responses are expected or evaluated. It is very important that the music therapist is trained to be empathic toward the needs and possibilities of the patient in order for the patient to experience being part of an interplay, where the feeling of isolation can be exceeded (14, 15). Finally, listening to music can support mood regulation and enhance quality of life (16–18).

We aimed to investigate whether the previous positive findings could be replicated in a Danish study sample. Utilizing a rigorous RCT design, we compared the efficacy of 25 weekly individual manualized music therapy sessions conducted by trained music therapists, adjunct to TAU, with an active control condition of 25 weekly individual manualized music listening sessions conducted by either social workers or care workers adjunct to TAU (12).

## Methods And Materials

Rationale and in-depth description of all study procedures were presented in a protocol publication (1).

### Trial Design

A multicenter, randomized, controlled, assessor- and patient-blinded parallel trial. The design included an active comparator including musical elements.

### Participants

Participants were all diagnosed with schizophrenia according to ICD-10, age 18–65 years, and with a minimum score of 4 on each of the following items of the Positive and Negative Syndrome Scale (PANSS): Blunted affect N1; Emotional withdrawal N2; Poor rapport N3; Passive/apathetic social withdrawal N4; Lack of spontaneity and flow of conversation N6 (19).

To ensure that negative symptoms were predominant, we excluded participants with secondary negative symptoms, as defined by a Calgary Depression Scale for Schizophrenia (CDSS) (20), i.e., a score above 7 on neurological or sedative side-effects; with a score above 1 on items 1.3 subjective sedation; 2.1 subjective muscle tension; 2.2 subjective rigidity; 2.3 subjective motor retardation; 2.5 tremor; or 2.6 subjective akathisia on the Task Force for Clinical Investigations (Udvalg for kliniske undersøgelser, UKU) side effects scale (21).

We also excluded participants with a first diagnosis of schizophrenia <2 years ago, hospitalization within the last 3 months before informed consent due to psychiatric illness, PANSS positive subscale (Delusions P1, Conceptual Disorganization P2, Hallucinatory behavior P3, Excitement P4, Grandiosity P5, Suspiciousness P6, and Hostility P7) above 28, change in psychotropic medication within the last month before informed consent, significant alcohol or drug abuse, which may interfere with study participation as judged by investigator; or those who had participated in individual music therapy within the last 2 months before informed consent.

Participants who had more than 30 days between two sessions or failed to attend more than five sessions were withdrawn from the study (22).

Participants from both hospital-based (out-patients) and social psychiatry from nine municipalities within the North Denmark Region and the Capital region of Denmark were included in the study.

### Interventions

All participants were offered 25 successive weekly sessions; either manualized music therapy or an active comparison condition with manualized music listening to selected playlists, termed intervention I and intervention II, respectively. Randomization was performed after baseline assessment. The protocol publication (1) states: “Due to the manual for intervention I, several music therapy methods and techniques can be applied, including active methods (where therapist and participant play or sing together, improvise, write songs or move to music) as well as receptive methods (where therapist and participant listen to music together either in the form of playlists selected by music therapists, or in the form of participant chosen or therapist chosen music). The focus of the manual is about perspectives on the therapist's way of being present and position in the therapeutic relationship. The manual for intervention II includes solely music listening to specific playlists developed by music therapists. This intervention is similar to one of the possible intervention methods in Intervention I and thus both intervention forms can be called music therapy activities and all performers of intervention I and II could be called therapists,” This overlap strengthened the blinding procedure.

Before enrolment, all participants were informed (1) that they would be offered 25 sessions of music therapy activities, (2) that two different activities would be tested, and (3) that those performing the sessions were all called therapists and were familiar with being together with people diagnosed with schizophrenia. At follow-up, 4 weeks after the end of the study, a short, semi-structured interview was performed with all participants, who had completed 25 sessions. All sessions took place in similarly equipped music therapy rooms in 10 locations, six in the North Denmark Region and four in the Capital Region of Denmark.

Intervention I was conducted by six trained and experienced music therapists (one male and five female therapists), familiar with the population under examination. Their background was a master's degree in music therapy and more than 1 year of clinical experience with the investigated population. They were also trained in, and followed, a manual developed for this study intervention. Several music therapy methods and techniques could be applied, including active methods (where therapist and participant play musical instruments, or sing together, improvise, write songs or move to music) as well as receptive methods (where therapist and participant listen to music together either in the form of playlists selected by music therapists, or in the form of music chosen by either the participant or the therapist). The manual focused on the therapist's way of being present and their positioning within the therapeutic relationship (23).

Intervention II was conducted by seven non-music therapists (four male and three female therapists), familiar with the diagnosis of the included population and also with knowledge or lived experience of people suffering from schizophrenia, either professionally (four had a profession within psychiatry e.g., social supporter) or privately [three had close contact (families and friends)], with the included population. These therapists were trained in and followed a manual developed for this intervention. Both manuals were developed according to unique, essential, possible and not-possible/proscribed principles for interventions and for the attitudes of the therapist working with this population (23, 24).

Intervention II activities consisted solely of listening to music from the app “The MusicStar” (25), containing a number of special playlists developed by music therapists inspired by a taxonomy identifying levels of intensity in recorded music (26). This activity was also included in the spectrum of possible activities in Intervention I.

Therapists in both interventions were unknown to the participant before the start of the intervention. Manuals for intervention I and II are available from the corresponding author.

### Outcome Measures

Participants were rated at baseline, after 15 sessions and after 25 sessions (end of study). Raters were blinded to treatment allocation at rating after 15 and 25 sessions of intervention I or II. All seven research assistants performing the rating procedure (project nurses and one medical student) were familiar with psychiatric patients. The primary outcome measure was change in PANSS Negative Subscale (items: blunted affect N1; emotional withdrawal N2; poor rapport N3; passive/apathetic social withdrawal N4, difficulty in abstract thinking N5, lack of spontaneity and flow of conversation N6, stereotyped thinking N7) from baseline to end of study. Secondary outcomes were defined as change in The Brief Negative Symptom Scale (BNSS) (27), WHOQOL-Bref (Quality of Life) (28), The Helping Alliance Questionnaire (Patient Version) (Haq-II) (29, 30), The Global Assessment of Functioning (GAF) (31).

The main hypothesis was that participants randomized to manualized music therapy sessions with an educated music therapist (Intervention I) adjunct to TAU would have a larger reduction of negative symptoms as compared to participants randomized to manualized music listening with a social or care worker (Intervention II) adjunct to TAU.

### Sample Size

Based on data from previous studies, we estimated an effect size of 0.6 (medium effect according to Cohen's *d*). With a selected level of significance of 0.05 and a power of 0.8, a power calculation performed in R with a conservative approach (applying a two-sided *t*-test with two samples), resulted in groups with a minimum of 44.5 persons. Previous studies utilized in the power calculation did not use an active control condition, which could result in a lower effect size in the current study. Furthermore, we estimated a drop-out between 10 and 30%, resulting in an estimated required sample size of 60 persons in each group.

### Randomization

Participants were randomized to groups of 2–4 by a researcher not involved in the study. Information on allocation to treatment Intervention I and II was sent by secure e-mail to the research coordinator, who initiated contact between the participant and the therapist responsible for the intervention to which the patient was allocated after meeting the inclusion criteria, and consent was provided.

### Blinding

Participants were blinded to the treatment allocation, and the research raters were blinded to the allocation of the participants they rated at baseline and after 15 and 25 sessions.

### Statistical Analysis

Categorical variables were summarized as counts and percentages while continuous variables were summarized as means with standard deviations.

For the intention-to-treat analysis, all available observations were used. For both primary and secondary outcomes, a mixed-effects model with treatment, time, and the interaction between treatment and time as fixed effects and participant as random effect, was used. An additive model (without the interaction term) was applied when the interaction between time and group was not significant. Every model was adjusted for the baseline score of the respective outcome variable.

An additional intention-to-treat analysis using only participants with no missing follow-up data was performed (completers analysis). In this difference in differences (DID) analysis the change from baseline to 25 weeks between the two groups was compared using ANCOVA to adjust for baseline outcome score.

To explore the impact of assumptions about missing data, we conducted a sensitivity analysis assuming no change for those who dropped out (last observation carried forward, LOCF).

The main analyses were carried out in Stata 16; additional analyses were conducted in R 4.0.5. Results with two-sided *p*-values <0.05 were considered statistically significant.

## Results

**Figure 1:**
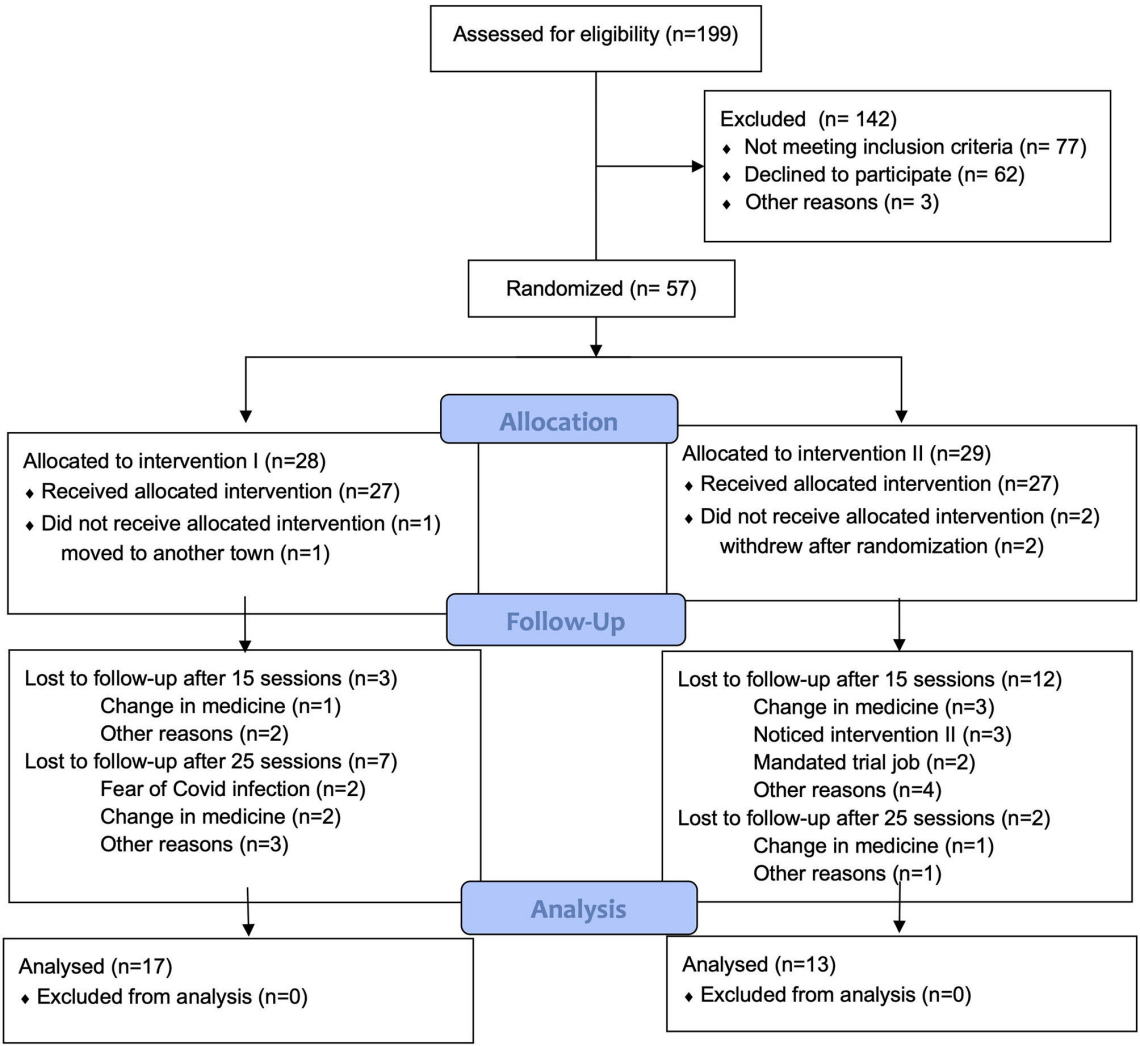
CONSORT 2010 flow diagram.

Recruitment started in March 2016 and was terminated in September 2019. Interventions were terminated in June 2020. During the trial period of 4 years and 3 months (from March 2016 to June 2020) 199 potential participants were screened with 57 randomized and 39 completing the study's initial 15 weeks, and 30 completing follow-up at 25 weeks, as shown in the flowchart (see Figure 1).

Duration of illness, age at inclusion, PANSS negative subscale, sex, substance misuse, schizophrenia subtype and education were similar in both interventional groups at baseline (Table 1).

**Table 1:** Demographics of study participants.

	**Music therapy**	**Music listening**	***p*-value**
	**(*n* = 28)**	**(*n* = 29)**	
**Sex^a^**			
*Male*	18 (66.7%)	16 (57.1%)	0.6
Age at baseline^b^	40.7 (13.2)	36.5 (11.3)	0.2
Duration of illness^b^	9.0 (7.9)	7.0 (8.7)	0.5
**Education^a^**			0.3
*Law-mandated school*	7 (25.0%)	6 (20.7%)	
*Grammar school (gymnasium or similar)*	8 (28.6%)	10 (34.5%)	
*Short vocation-oriented courses*	5 (17.9%)	2 (6.9%)	
*Vocational/apprenticeship training*	4 (14.3%)	8 (27.6%)	
*University training*	1 (3.6%)	3 (10.3%)	
*Other*	1 (3.6%)	-	
**Schizophrenia subtype^a^^,^ ^c^**			0.6
*Paranoid Schizophrenia*	17 (73.9%)	19 (70.4%)	
*Hebephrenic schizophrenia*	1 (4.3%)	-	
*Undifferentiated schizohprenia*	3 (13.0%)	5 (18.5%)	
*Simple schizophrenia*	1 (4.3%)	-	
*Remaining subtypes*	1 (4.3)	3 (11.1%)	
Misuse of alcohol or substances^a^	1 (3.6%)	1 (3.4%)	>0.9

a *Frequency (%); p-value from Fisher's exact-test*.

b *Mean (SD); p-value from two-sided t-test*.

c *Schizophrenia subtype data were missing for 7 participants*.

At baseline no differences were observed between groups on PANSS total score, PANSS subscale scores, BNSS total score and BNSS subscale scores, CDSS, GAF, WHO-QoL total score, and HAQ II total score (Table 2).

**Table 2:** Baseline scores of study participants.

	**Intervention I**		**Intervention II**	
	**Mean**	**95% CI**	**Mean**	**95% CI**
PANSS total	73.9	(69.0–78.8)	68.6	(65.1–72.1)
PANSS negative subscale	23.4	(21.7–25.1)	23.4	(21.4–25.4)
PANSS positive subscale	15.6	(13.6–17.7)	13.7	(12.3–15.1)
PANSS general subscale	34.9	(31.8–38.0)	31.5	(29.2–33.8)
BNSS total	37.4	(33.9–41.0)	36.2	(32.4–40.0)
BNSS anhedonia subscale	10.4	(9.4–11.4)	10.2	(9.0–11.4)
BNSS distress subscale	2.4	(1.8–2.9)	1.6	(0.9–2.2)
BNSS Asociality subscale	5.5	(4.6–6.3)	5.2	(4.5–5.9)
BNSS Avolition subscale	6.5	(5.8–7.1)	6.5	(5.7–7.2)
BNSS Blunted affect subscale	7.9	(6.6–9.1)	7.9	(6.4–9.5)
BNSS Alogia subscale	4.9	(3.8–6.1)	4.9	(3.9–5.8)
Calgary Depression Scale for Schizophrenia	3.2	(2.1–4.3)	3.7	(2.9–4.4)
GAF	39.4	(36.4–42.3)	41.3	(38.7–43.8)
WHO-QoL total score	77.0	(71.8–82.1)	76.8	(71.8–81.8)
WHO-QoL Physical health domain raw score	20.8	(18.8–22.7)	21.9	(19.9–23.9)
WHO-QoL Psychological domain raw score	15.6	(13.9–17.4)	14.3	(12.6–15.9)
WHO-QoL Social Relationships domain raw score	9.0	(8.4–9.7)	9.4	(8.5–10.3)
WHO-QoL Environment domain raw score	24.9	(22.9–26.8)	26.0	(24.6–27.3)
HAQ II Helping Alliance Questionnaire	5.0	(4.6–5.3)	4.7	(4.4–5.1)

*PANSS, Positive and Negative Symptom Scale; BNSS, Brief Negative Symptom Scale; GAF, Global Assessment of Functioning; WHO-QoL, World Health Organization Quality of Life; HAQ II, Helping Alliance Questionnaire (Patient version II; completed after 5 sessions); CI, Confidence interval*.

**Figure 2:**
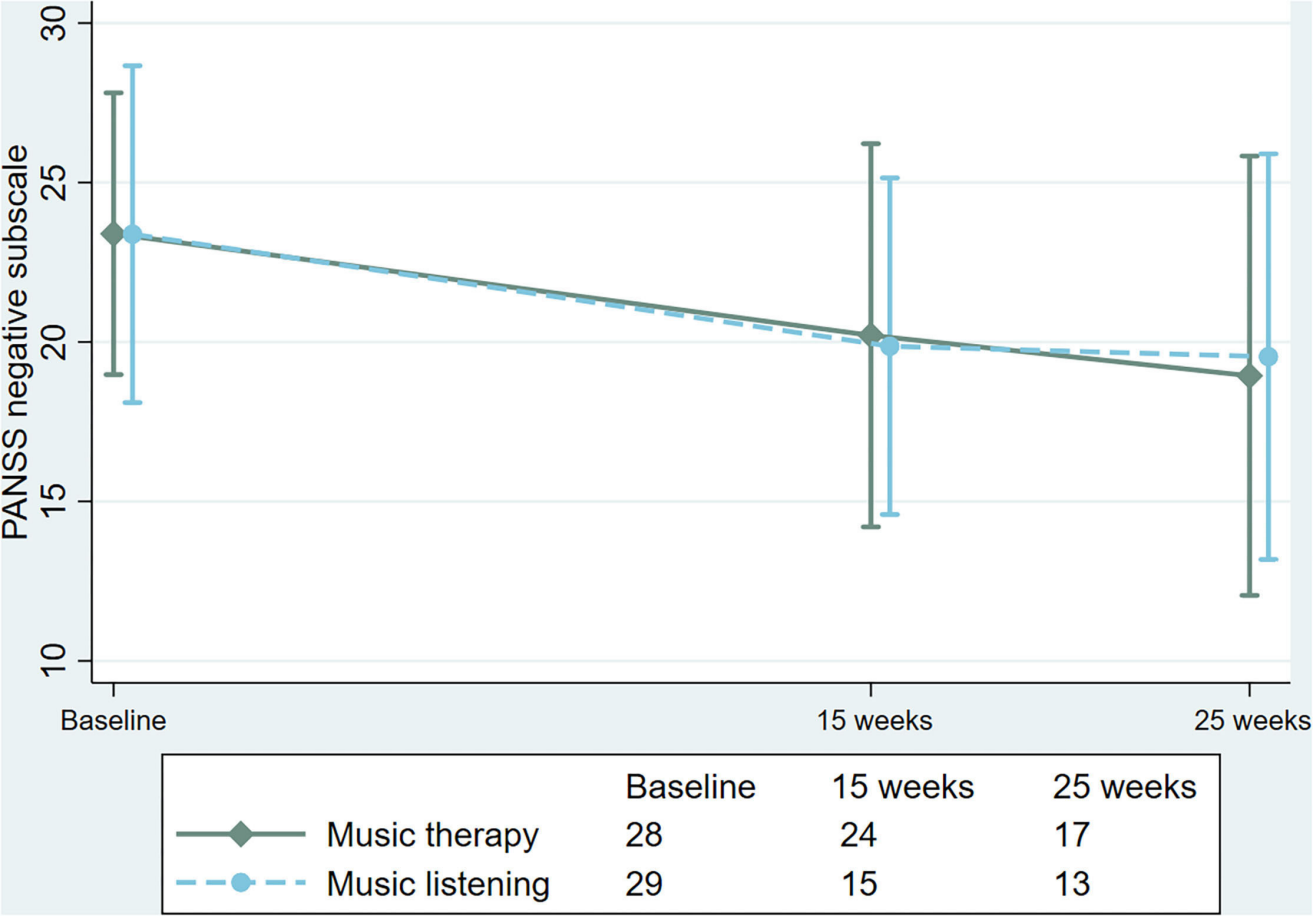
Changes in Positive and Negative Syndrome Scale (PANSS), negative subscale in the intention to treat population.

**Figure 3:**
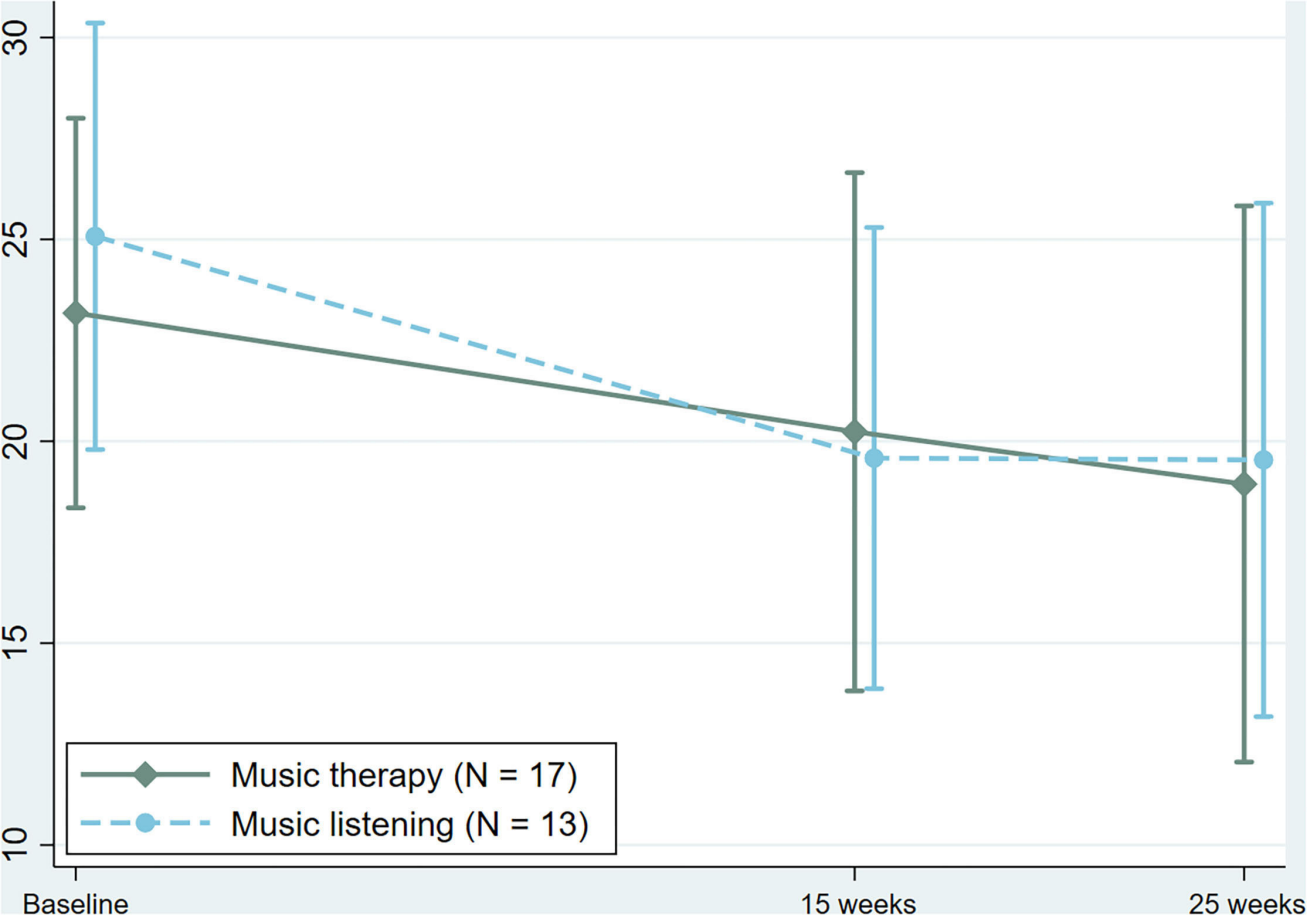
Changes in Positive and Negative Syndrome Scale (PANSS), negative subscale in the completer's population.

For the primary outcome of the PANSS negative subscale (across both follow-up time points), there was no significant interaction between treatment and time and no significant difference was observed between groups, with a coefficient of −0.24 (95% CI −1.76 to 1.27, *P* = 0.754) in the intention to treat analysis, and a coefficient of −0.98 (95% CI −5.06 to 3.09, *P* = 0.625) when only analyzing completers. Mean and standard deviation for each time-point measured depicted in Figures 2, 3 for intention to treat as well as completers, respectively. The additional sensitivity analysis using LOCF showed similar results and no significant difference between groups.

On secondary outcomes, no significant interactions between treatment and time were found and no significant difference between group differences were observed in The Brief Negative Symptom Scale (BNSS), WHOQOL-Bref (Quality of Life, total score), The Helping Alliance Questionnaire (Patient Version) (Haq-II) and The Global Assessment of Functioning (GAF) in the intention to treat or completers populations (Table 3).

**Table 3:** Between group differences for intention to treat and completers for secondary outcomes.

	**Intention to treat**		**Completers**	
**Between group comparison**	**Difference**	**95% CI**	**Difference**	**95% CI**
BNSS total score	−0.4	(−3.0–2.1)	−2.5	(−10.7–5.6)
WHOQOL total score	−0.9	(−4.0–2.3)	−4.9	(−14.0–4.3)
HAQ II	−0.1	(−0.2–0.1)	−0.3	(−0.8–0.2)
GAF	1.4	(−0.8–3.5)	1.9	(−5.5–9.2)

*Results of the primary outcome, see text*.

*BNSS, Brief Negative Symptom Scale; WHO-QoL, World Health Organization Quality of Life; HAQ II, Helping Alliance Questionnaire (Patient version II); GAF, Global Assessment of Functioning; CI, Confidence interval*.

Interestingly, significant differences were observed from baseline to end of study within groups (group I, *P* = 0.002); (group II; *P* = 0.001).

A single adverse event was reported during the trial, as a participant randomized to intervention II had stopped taking medication and came to a rating procedure in a state resulting in hospitalization. Fourteen participants were excluded from the study due to protocol violation, change in medicine (eight participants), and/or too much absence (six participants). Two participants withdrew consent due to dissatisfaction with the first rating procedure. Other dropout reasons were: identification of being allocated to intervention II (three participants), forced to trial job by the system (two participants), fear of COVID-19 (two participants), moving to another town (one participant), and without a specified reason (three participants). A larger proportion of patients randomized to intervention II dropped out before the primary assessment (week 15), with similar drop-out rates between groups at weeks 25 (Figure 1).

## Discussion

This study is the first Danish trial of music therapy for patients diagnosed with schizophrenia, and also one of the first international studies with an active control condition including music, and with both assessor and participant blinding. No between groups difference of music therapy vs. music listening on negative symptoms were found on the primary outcome. However, participants in both music therapy and music listening had a reduction in PANSS negative subscale score over the study period, with the reduction of negative symptoms within both groups from baseline to termination being of a clinically relevant size. This finding must be interpreted with caution, as it may be a result of baseline inflation on the primary outcome, as well as a possible Hawthorne effect.

In the present study we did not find between groups difference on secondary outcomes as measured by The Brief Negative Symptom Scale (BNSS), WHOQOL-Bref (Quality of Life, total score), The Helping Alliance Questionnaire (Patient Version) (Haq-II) and The Global Assessment of Functioning (GAF), similar to the findings on the primary outcome measure of PANSS negative subscale.

### Comparison With Other Studies

Previous studies are not directly comparable to the present, as the primary intervention in most studies was group music therapy, not individual music therapy (32–37). Most studies have investigated music therapy as compared to TAU, without an active control condition (3, 5, 6, 9, 34, 36), resulting in an increased risk of a measured Hawthorne effect. In the present study, we chose to include patients with a minimal score on the PANSS positive subscale, and a high score for the negative subscale, excluding patients with depression or side-effects, which could result in a higher score on a scale measuring negative symptoms. This is in contrast to previous studies, in which illness severity was divided into mild, moderate or severe dependent on the total score of the symptoms under investigation, with the majority of studies being conducted with patients with mild to moderate symptom severity (5).

Furthermore, the choices regarding the music therapy intervention itself differs in part from previous studies, where trained music therapists performed the treatment in some, but not all studies. This is relevant in the present study because both intervention I and intervention II had musical elements, but intervention I was conducted by trained music therapists. In this respect, the control group condition here seems similar to the experimental condition in other studies (5, 33, 38). Since no difference was observed between groups in this study, both interventions may be beneficial for the participants, albeit we are unable to disentangle a true effect of the intervention from a possible indirect effect of participating in a trial, e.g., the Hawthorne effect or a possible baseline inflation of the PANSS negative subscale score. The results can be seen in relation to the fact that, in later years, staff members in hospital psychiatry in Denmark have been trained by music therapists in applying the app “The Music Star” (25) to daily routines. The app is user friendly and presents a number of playlists with music pieces ranging from low to moderate intensity, indicated by colors ranging from light blue to dark red. This study indicates that staff training may be of benefit not only to inpatients but also to outpatients living with schizophrenia, with predominantly negative symptoms.

Our findings clearly showed no difference between groups and there can be different reasons for that, the explanations of which will be beyond the scope of this study. We can suggest that future studies examine if subgroups within this population may benefit from a predominantly controlled music therapy approach or from a music therapy approach tailored to the needs of the specific client.

### Primary and Secondary Symptoms

Negative symptoms can conceptually be defined as primary or secondary, and in the present study the CDSS and UKU were administered at baseline to minimize the risk of participants included predominantly presenting with secondary negative symptoms, such as additional depression or side effects from antipsychotic medicine, as proposed in several meta-analyses (4, 5, 9).

### Application of Measurement Tools

The number of measurement tools for each rating session could be seen as ambitious and as a limitation for recruiting participants from this population. The participants were informed that the rating session would take approximately one and a half hours and that a video recording would be a small part of the session. This information may have resulted in anxiety concerning the rating procedure, resulting in lack of attendance. Ethically we intended to make the information procedure as clear and transparent as possible and for the rating procedures we only employed research-assistants familiar with psychiatric patients. A recent scoping review of the use of music in mental health focusing on severe mental illnesses (SMI) including 349 studies reports that most studies referred to use of music with people living from schizophrenia. The review emphasizes, as one of five recommendations for future studies, that researchers studying the use of music in mental health “consider developing core outcome sets and core measures” in their studies. This is because an existing heterogeneity in review studies “often limits the combination and comparison of the results of individual studies” [(39), p. 15]. In this study, we met these criteria by applying core outcome sets developed for the investigated exact symptoms in schizophrenia.

In the recently released EPA guidelines on treatment of negative symptoms in schizophrenia it is emphasized that “further evidence is needed to formulate sound recommendation for primary, persistent negative symptoms” [(40), p. 1].

### Inpatients vs. Outpatients

The EPA guidelines recommend that “an access to treatment and to psychosocial rehabilitation should be ensured for patients with negative symptoms.” Music therapy is mentioned as one possibility with the following request; “music therapy seems to have a potential for improving negative symptoms, but a larger trial including outpatients would be needed before a recommendation can be given” [(40), p. 10]. In the present study all 57 included participants were outpatients.

### Strengths and Limitations

The most important limitations of the study are its limited sample size and high attrition. While we did analyse all available data in the intention-to-treat analysis, it may have been possible to retain more participants by expanding the follow-up period. However, we had to restrict the follow-up period by a reasonable time, and it is unlikely that participants who stopped attending sessions would have been available later. Furthermore, patients were not allowed to change primary antipsychotic medication, as this could potentially influence primary and secondary negative symptoms, and as a result participants discontinued the study. To address the problem of attrition, we conducted a sensitivity LOCF analysis where all participants randomized were included; the results of that analysis did not change the conclusions.

Secondly, data show a larger number of dropouts in Intervention II as compared to I at primary endpoint, but with a larger dropout in Intervention I as compared to Intervention II from 15 weeks to the end of follow-up, at 25 weeks. In general, attrition was higher in Intervention II–55%, compared to 39% in Intervention I at week 15—but was high across groups. This may be a consequence of the vulnerability of the population under investigation, resulting in survival bias, with most dropouts early, also known as the positive adherence effect, although this would only account for the observation in invention II. The pattern of differences between Intervention I and II could also be driven by differences in how interventions were perceived, but is likely by chance, with similar reasons for dropout in the two groups at week 25 (see Figure 1, Flow Chart). Three participants from group II expressed that they were aware that they did not attend “real music therapy” (as one participant described it), and therefore withdrew consent (see Figure 1). Figure 1 also shows a large proportion of potential participants initially showing interest and wanting to participate, but not turning up for the first rating procedure, but with only three participants in total not being allocated. In the study population consisting of patients with primarily negative symptoms, social isolation and lack of initiative are common and may have contributed to the low attendance for the first rating. When interpreting these results, the relatively small sample size and high attrition must therefore be considered: 27 of 57 participants (47%) were lost to the last follow-up; some due to the societal lock-down as a result of COVID-19.

Another limitation of the study is its reliance on the original scoring method of the PANSS, as presented by Kay et al. in 1987 (19). More recent work has presented different scoring methods with different definitions of subscales across different samples [for an overview and emerging consensus see (41)]. The large number of different models highlights an ongoing discussion about the nature and scope of the negative symptom cluster. In the present clinical trial, it was important to analyze the data according to the pre-specified protocol and analysis plan to avoid false positive findings. However, the factor structure and inter-relation between different symptoms in the PANSS scale should be further explored in future work.

The ambition in the present study was to apply a rigorous research design with manualized interventions, standardized outcomes and an active control condition, in order to reduce the risk of observing Hawthorne effects when studying adjunct music therapy. We further established a blinded condition and ensured that all potential participants received the same information and expectations (all intervention agents were called “therapists,” all sessions took place in well-equipped music rooms—the same rooms for both conditions and music was a part of both conditions and one music activity overlapped the two interventions). Nevertheless, three participants in the control group detected which group they were allocated to—and consequently withdrew consent to participate. The remaining participants did not express any such knowledge—not even in the interview situation, where they were encouraged to ask questions or give comments of whatever character, suggesting a high degree of blinding, which further supported the design. An important limitation of this study is the small sample size and high drop-out rate, which could result in existing effects not being detected due to a Type II error. The estimated 60 participants in both groups could not be reached due to severe recruitment problems.

## Conclusion

In conclusion, no difference between groups randomized to music therapy vs. musical listening and rated in a blinded manner was observed during the study period, resulting in no clear recommendation for which intervention to use as first choice in therapy with patients diagnosed with schizophrenia and suffering from predominantly negative symptoms.

## Data Availability Statement

The raw data supporting the conclusions of this article will be made available by the authors, without undue reservation.

## Ethics Statement

The studies involving human participants were reviewed and approved by the Regional Committee on Biomedical Research Ethics, the North Denmark Region. The patients/participants provided their written informed consent to participate in this study.

## Author Contributions

IP, JN, and LB: conceptualization and investigation. IP, JN, LB, LR, NH, and SJ: methodology. CG, JN, and LR: software. IP, LR, and SJ: resources and data curation. IP and RN: writing—original draft preparation. CG, IP, JA, JN, LB, LR, NH, and RN: writing—review and editing. IP, JA, JN, and RN: supervision. IP: project administration and funding acquisition. All authors contributed to the article and approved the submitted version.

## Funding

We gratefully thank the TRYG Foundation (ID 100977) and the Obel Family Foundation for the funding of the study. We also acknowledge Aalborg University, Department of Communication and Psychology and Aalborg University Hospital, Department of Psychiatry for their joint financial support in this study.

## Conflict of Interest

LR is co-owner of the design rights for the MusicStar app. The MusicStar application was developed in 2015-2016 in a joint-venture between Aalborg University Hospital, Psychiatry, the private company AudioCura Aps., and music therapists Helle Nystrup Lund and Lars Rye Bertelsen, who are co-owners of the design rights to the app. RN has received research grants from H. Lundbeck and Otsuka Pharmaceuticals for clinical trials, received speaking fees from Bristol-Myers Squibb, Astra Zeneca, Janssen & Cilag, Lundbeck, Servier, Otsuka Pharmaceuticals, Teva A/S and Eli Lilly and has acted as advisor to Astra Zeneca, Eli Lilly, Lundbeck, Otsuka Pharmaceuticals, Takeda and Medivir, and has acted as investigator for Janssen-Cilag, Lundbeck, Boehringer, Compass and Sage. The remaining authors declare that the research was conducted in the absence of any commercial or financial relationships that could be construed as a potential conflict of interest.

## Publisher's Note

All claims expressed in this article are solely those of the authors and do not necessarily represent those of their affiliated organizations, or those of the publisher, the editors and the reviewers. Any product that may be evaluated in this article, or claim that may be made by its manufacturer, is not guaranteed or endorsed by the publisher.
